# Supplementary material for: Unveiling the Bioactive Potential of the Invasive Jellyfish Phyllorhiza punctata Through Integrative Transcriptomic and Proteomic Analyses
Source: Biomolecules. 2025 Aug 4;15(8):1121. doi: 10.3390/biom15081121 (PMC12383608; doi:10.3390/biom15081121)
Supplement: Supplementary file 1 [file biomolecules-15-01121-s001.zip › Suplementary Material/FileS3/PEPFOLD4/XP_065060405_8378/clusters.html]

 PEP-FOLD clusters


Download archive of all models  here   

|  |  |  |
| --- | --- | --- |
| # | file | sOPEP |
| # cluster 1 - Size: 34 |  |  |
|  | XP\_065060405\_8378-model1 | -19.6324 |
|  | XP\_065060405\_8378-model1.1 | -19.6324 |
|  | XP\_065060405\_8378-model1.2 | -19.6324 |
|  | XP\_065060405\_8378-model1.3 | -19.6324 |
|  | XP\_065060405\_8378-model1.4 | -19.6324 |
|  | XP\_065060405\_8378-model1.5 | -19.6324 |
|  | XP\_065060405\_8378-model1.6 | -19.6324 |
|  | XP\_065060405\_8378-model1.7 | -19.6324 |
|  | XP\_065060405\_8378-model1.8 | -19.409 |
|  | XP\_065060405\_8378-model1.9 | -19.409 |
|  | XP\_065060405\_8378-model1.10 | -19.409 |
|  | XP\_065060405\_8378-model1.11 | -19.409 |
|  | XP\_065060405\_8378-model1.12 | -19.409 |
|  | XP\_065060405\_8378-model1.13 | -19.409 |
|  | XP\_065060405\_8378-model1.14 | -19.409 |
|  | XP\_065060405\_8378-model1.15 | -19.409 |
|  | XP\_065060405\_8378-model1.16 | -19.409 |
|  | XP\_065060405\_8378-model1.17 | -19.409 |
|  | XP\_065060405\_8378-model1.18 | -19.409 |
|  | XP\_065060405\_8378-model1.19 | -19.409 |
|  | XP\_065060405\_8378-model1.20 | -19.409 |
|  | XP\_065060405\_8378-model1.21 | -19.409 |
|  | XP\_065060405\_8378-model1.22 | -19.409 |
|  | XP\_065060405\_8378-model1.23 | -19.409 |
|  | XP\_065060405\_8378-model1.24 | -19.4032 |
|  | XP\_065060405\_8378-model1.25 | -19.4032 |
|  | XP\_065060405\_8378-model1.26 | -19.3993 |
|  | XP\_065060405\_8378-model1.27 | -19.3993 |
|  | XP\_065060405\_8378-model1.28 | -19.3146 |
|  | XP\_065060405\_8378-model1.29 | -19.3146 |
|  | XP\_065060405\_8378-model1.30 | -19.3146 |
|  | XP\_065060405\_8378-model1.31 | -19.311 |
|  | XP\_065060405\_8378-model1.32 | -19.2984 |
|  | XP\_065060405\_8378-model1.33 | -19.2672 |
| # cluster 2 - Size: 18 |  |  |
|  | XP\_065060405\_8378-model2 | -19.5535 |
|  | XP\_065060405\_8378-model2.1 | -19.5105 |
|  | XP\_065060405\_8378-model2.2 | -19.5105 |
|  | XP\_065060405\_8378-model2.3 | -19.5105 |
|  | XP\_065060405\_8378-model2.4 | -19.5105 |
|  | XP\_065060405\_8378-model2.5 | -19.5105 |
|  | XP\_065060405\_8378-model2.6 | -19.5105 |
|  | XP\_065060405\_8378-model2.7 | -19.5105 |
|  | XP\_065060405\_8378-model2.8 | -19.4614 |
|  | XP\_065060405\_8378-model2.9 | -19.4039 |
|  | XP\_065060405\_8378-model2.10 | -19.4039 |
|  | XP\_065060405\_8378-model2.11 | -19.4039 |
|  | XP\_065060405\_8378-model2.12 | -19.4039 |
|  | XP\_065060405\_8378-model2.13 | -19.3766 |
|  | XP\_065060405\_8378-model2.14 | -19.341 |
|  | XP\_065060405\_8378-model2.15 | -19.341 |
|  | XP\_065060405\_8378-model2.16 | -19.341 |
|  | XP\_065060405\_8378-model2.17 | -19.2239 |
| # cluster 3 - Size: 6 |  |  |
|  | XP\_065060405\_8378-model3 | -19.5118 |
|  | XP\_065060405\_8378-model3.1 | -19.5118 |
|  | XP\_065060405\_8378-model3.2 | -19.3899 |
|  | XP\_065060405\_8378-model3.3 | -19.3781 |
|  | XP\_065060405\_8378-model3.4 | -19.3649 |
|  | XP\_065060405\_8378-model3.5 | -19.3346 |
| # cluster 4 - Size: 12 |  |  |
|  | XP\_065060405\_8378-model4 | -19.5023 |
|  | XP\_065060405\_8378-model4.1 | -19.5023 |
|  | XP\_065060405\_8378-model4.2 | -19.5023 |
|  | XP\_065060405\_8378-model4.3 | -19.5023 |
|  | XP\_065060405\_8378-model4.4 | -19.5023 |
|  | XP\_065060405\_8378-model4.5 | -19.4726 |
|  | XP\_065060405\_8378-model4.6 | -19.4726 |
|  | XP\_065060405\_8378-model4.7 | -19.4074 |
|  | XP\_065060405\_8378-model4.8 | -19.4074 |
|  | XP\_065060405\_8378-model4.9 | -19.3845 |
|  | XP\_065060405\_8378-model4.10 | -19.2443 |
|  | XP\_065060405\_8378-model4.11 | -19.2443 |
| # cluster 5 - Size: 5 |  |  |
|  | XP\_065060405\_8378-model5 | -19.4617 |
|  | XP\_065060405\_8378-model5.1 | -19.4427 |
|  | XP\_065060405\_8378-model5.2 | -19.4427 |
|  | XP\_065060405\_8378-model5.3 | -19.3306 |
|  | XP\_065060405\_8378-model5.4 | -19.3271 |
| # cluster 6 - Size: 11 |  |  |
|  | XP\_065060405\_8378-model6 | -19.4389 |
|  | XP\_065060405\_8378-model6.1 | -19.4389 |
|  | XP\_065060405\_8378-model6.2 | -19.4389 |
|  | XP\_065060405\_8378-model6.3 | -19.4389 |
|  | XP\_065060405\_8378-model6.4 | -19.4389 |
|  | XP\_065060405\_8378-model6.5 | -19.4389 |
|  | XP\_065060405\_8378-model6.6 | -19.4335 |
|  | XP\_065060405\_8378-model6.7 | -19.4335 |
|  | XP\_065060405\_8378-model6.8 | -19.3663 |
|  | XP\_065060405\_8378-model6.9 | -19.3663 |
|  | XP\_065060405\_8378-model6.10 | -19.3663 |
| # cluster 7 - Size: 9 |  |  |
|  | XP\_065060405\_8378-model7 | -19.4071 |
|  | XP\_065060405\_8378-model7.1 | -19.4071 |
|  | XP\_065060405\_8378-model7.2 | -19.4071 |
|  | XP\_065060405\_8378-model7.3 | -19.3804 |
|  | XP\_065060405\_8378-model7.4 | -19.3593 |
|  | XP\_065060405\_8378-model7.5 | -19.3593 |
|  | XP\_065060405\_8378-model7.6 | -19.3593 |
|  | XP\_065060405\_8378-model7.7 | -19.3556 |
|  | XP\_065060405\_8378-model7.8 | -19.2268 |
| # cluster 8 - Size: 4 |  |  |
|  | XP\_065060405\_8378-model8 | -19.3972 |
|  | XP\_065060405\_8378-model8.1 | -19.3972 |
|  | XP\_065060405\_8378-model8.2 | -19.3405 |
|  | XP\_065060405\_8378-model8.3 | -19.3405 |
| # cluster 9 - Size: 1 |  |  |
|  | XP\_065060405\_8378-model9 | -19.2516 |
|  |  |  |
